# Predicted Global Redistribution of *Lagria nigricollis* (Coleoptera: Tenebrionidae) Under Future Climate Change

**DOI:** 10.3390/insects16121227

**Published:** 2025-12-03

**Authors:** Manlu Zhao, Jieqiong Wang, Fen Liu, Yunchun Li, Hanlan Fei, Zhonghua Wei, Aimin Shi

**Affiliations:** The Key Laboratory of Southwest China Wildlife Resources Conservation of the Ministry of Education, College of Life Sciences, China West Normal University, Nanchong 637009, China; zmlzjp999@163.com (M.Z.); jqwang0902@126.com (J.W.); liufen2027@163.com (F.L.); liyc2260@cwnu.edu.cn (Y.L.); feihanlan@163.com (H.F.); aiminshi2003@126.com (A.S.)

**Keywords:** darkling beetles, Lagriinae, MaxEnt, suitable area

## Abstract

*Lagria nigricollis* (Coleoptera: Tenebrionidae) is a forest pest, widely distributed in East Asia. The impact of climate change on its distribution is currently unknown. To better understand how the geographic distribution of this species responds to future climate change, this study employed the MaxEnt model, integrating 21 environmental variables, to explore changes in its distribution range under different climate scenarios. The results indicated that the average area under the receiver operating characteristic curve (AUC) of the MaxEnt model across different periods is 0.991, demonstrating the reliability of the model’s predictions. The precipitation of the warmest quarter (bio18) and the temperature seasonality (bio04) were the most important environmental variables. Under current conditions, the suitable areas for *L. nigricollis* are mainly located in China (North China and Central China), the Korean Peninsula, and Japan (Kyushu, Shikoku, and the southern part of Honshu). In the 2050s and 2070s, suitable areas are expected to expand under three socioeconomic pathways, which are 1.08–1.33 times larger than they are currently. The expanded areas are mainly located in the northern part of the current suitable regions. The centroids of suitable areas will shift northward under future climate conditions. To limit the northward expansion of this species, primary control regions will focus on China (the junction of Inner Mongolia–Xinjiang and Northern China), Russia (the southeastern part of the Far Eastern Federal District), and Japan (Hokkaido). The results of this study provide a scientific basis for the forest pest control and distribution prediction of *Lagria nigricollis.*

## Simple Summary

Climate change has significantly impacted species diversity and distribution. However, research on its effects on the distribution of Tenebrionidae species remains limited. *Lagria nigricollis* Hope, 1843, an important member of Tenebrionidae, is widely distributed in East Asia. The adults of this species feed on the leaves of mulberry, locust, and maple trees, and are considered forest pests. This study utilizes the maximum entropy (MaxEnt) model to assess the potential impact of future climate change (2041–2060, and 2061–2080) on the distribution of *L. nigricollis* under three socioeconomic pathways (SSP1-2.6, SSP2-4.5, and SSP5-8.5). The results suggest that the suitable areas for *L. nigricollis* are currently located in China (North China, Central China, and Northeast China), the Korean Peninsula, and Japan. In the 2050s and 2070s, the suitable areas are expected to expand and shift northward. At present, the primary management regions for this species are located in China (Northern China, Central China, and Northeast China), the Korean Peninsula, and Japan (Kyushu, Shikoku, and southern Honshu).

## 1. Introduction

Global warming is one of the major scientific challenges facing ecosystems worldwide, and it has had a significant impact on biodiversity [1]. The rapid changes in climate and habitat loss have profoundly affected species distribution and the maintenance of biodiversity [2,3]. For forest ecosystems, climate-driven shifts in pest species distributions have become a critical concern, posing significant risks to forest health and sustainable forestry production.

The family Tenebrionidae is species-rich, with 11 subfamilies and approximately 30,000 species, making it one of the largest families in Coleoptera. Its feeding habits are diverse, including mycophagy, herbivory, omnivory, and carnivory. Some species in this family are considered pests due to their direct or indirect economic impact [4]. *Lagria nigricollis* Hope, 1843 is an important member of Tenebrionidae. It is a forest pest, with adults feeding on the leaves of various trees such as mulberry and locust, causing leaf damage, hindering tree growth, and making the trees more susceptible to secondary pests and diseases. This poses a significant threat to forestry production and forest ecosystem health [5]. Global warming may lead to the expansion and shift in its suitable distribution range, intensifying the risk of damage. Therefore, studying the evolution of this species’ distribution patterns under climate change can provide scientific support for precise pest control and disaster prediction, which is of significant practical value for ensuring the safety of forestry resources and maintaining ecological balance.

Species distribution models (SDMs) have emerged to study the relationships between species distribution and environmental variables. Several models have been proposed and widely applied in ecology, evolution, conservation, invasive-species management, and other fields [6,7,8,9,10], including Maximum Entropy (MaxEnt) Modeling [11], ENphylo [12], the Bioclimate Analysis and Prediction System (BIOCLIM) [13], and CLIMEX [14], among others. Compared to other models, MaxEnt offers high simulation accuracy and is less sensitive to sample size, performing especially well in cases with small sample sizes [15,16,17]. Therefore, it is widely used to predict the potential distribution areas of animals and plants [18,19,20,21,22]. However, this species is widely distributed in East Asia [23,24]. Studies on this species are currently at the taxonomic stage [23,24,25]. However, no research has been conducted on the impact of climate change on its distribution range. In Tenebrionidae, only one species, *Luprops orientalis* (Motschulsky, 1868), has been reported to have a relationship between its geographic distribution and climatic factors [26]. This severely hinders the understanding of the adaptive patterns of tenebrionid species in response to climate change.

To explore the impact of climate change on the distribution of *Lagria nigricollis*, this study used MaxEnt modeling, combined with 21 environmental variables, to predict the changes in its suitable habitat under climate change. This species has a broad distribution, and studying the effects of climate change on its suitable habitat is crucial for better understanding how widely distributed tenebrionid species respond to future climate change.

## 2. Materials and Methods

### 2.1. Obtaining and Processing Occurrence Data

**Figure 1:**
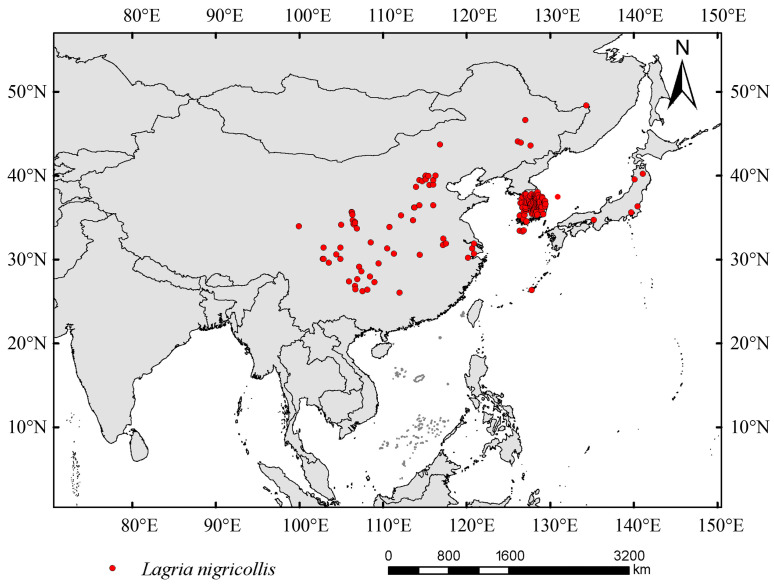
The distribution of *Lagria nigricollis*.

The occurrence data of *Lagria nigricollis* were obtained from the Global Biodiversity Information Facility (GBIF, https://www.gbif.org/, accessed on 10 July 2025) [27], the China National Knowledge Infrastructure (CNKI, https://www.cnki.net/, accessed on 23 July 2025), and the National Animal Collection Resource Center (http://museum.ioz.ac.cn/, accessed on 23 July 2025). To ensure data quality and the reliability of model predictions, this study processed the data based on SDM. Firstly, occurrence data recorded only at the provincial, autonomous region, or county level (without latitude and longitude) were excluded, as such vague data can affect the accuracy of SDMs [28]. Secondly, duplicate points were manually removed using Microsoft Excel, with latitude and longitude coordinates as the core criteria for determination. And redundant distribution points within the same grid were removed using ArcGIS v10.2. Finally, ENMTools v1.1.5 software was used for further screening. Based on a 2.5 arc-minute environmental data resolution, a 5 km spatial buffer distance was set. Representative points within the clustered areas were retained according to the principle of “maximizing environmental heterogeneity,” effectively eliminating issues such as model performance overestimation, misjudgment of variable importance, and prediction bias caused by spatial clustering effects. After these steps, 234 initial points were retained, and ultimately, 185 high-quality distribution records were obtained for suitable habitat prediction (Figure 1).

### 2.2. Environmental Variables

Compared to other variables, bioclimatic and soil variables are the more important factors affecting SDMs [29]. There were 21 environmental variables used for MaxEnt modeling, including bioclimate variables, elevation, global land cover type, and vegetation. The bioclimate variables were obtained from WorldClim (http://www.worldclim.org/, accessed on 27 July 2025), with a 2.5 arc-minute spatial resolution, which was chosen based on the strong dispersal capacity of *Lagria nigricollis*. The global land cover type v3 and vegetation v2 were downloaded from GitHub (https://globalmaps.github.io/, accessed on 27 July 2025) [30]. The elevation data was downloaded from EarthEnv (https://www.earthenv.org/, accessed on 27 July 2025), with coverage of about 91% of the globe [31]. The environmental variables include both current (1970–2000) and future (2041–2060, 2061–2080) climate projections. The future projections were obtained from the BCC-CSM2-MR model within the framework of the Coupled Model Intercomparison Project Phase 6 (CMIP6) [32,33]. This model has demonstrated high simulation accuracy for the East Asia region and is capable of accurately capturing key climatic features. The processing workflow is as follows: the NetCDF raw data from the BCC-CSM2-MR model were parsed, target variables were extracted, and invalid values were handled. Spatial resolution was unified through bilinear interpolation, and the data were aggregated to an annual scale. Outliers were detected and corrected using the boxplot method. After Z-score normalization, the data were output in ASCII format suitable for the MaxEnt modeling. Three shared socioeconomic pathways (SSPs) were selected for modeling: the low-emission scenario (SSP1-2.6), the intermediate-emission scenario (SSP2-4.5), and the high-emission scenario (SSP5-8.5). Specifically, SSP1-2.6 represents a sustainable development pathway with rapid emission reduction to achieve net-zero greenhouse gas (GHG) emissions by the end of the century. SSP2-4.5 reflects a middle-of-the-road development trajectory, with GHG emissions peaking around mid-century and stabilizing thereafter. SSP5-8.5 corresponds to a fossil fuel-intensive development pathway with continuous growth in GHG emissions, resulting in the highest radiative forcing by 2100.

A Pearson correlation analysis was conducted on the environmental variables to avoid errors from high autocorrelation (Figure S1), with only the most strongly correlated variable removed for groups with |r| > 0.85. The environmental variables were then filtered using PCA transformation. Finally, 10 variables were selected for the modeling test (Table 1), and their contributions to the climate suitability model were determined using Worthington’s method [34]. This method is a widely used quantitative tool for evaluating variable importance in ecological niche modeling. It calculates the relative contribution of each environmental variable by sequentially excluding individual variables and measuring the degree of subsequent model performance degradation.

### 2.3. Modeling Methods

The MaxEnt v3.4.1 and ArcGIS v10.2 were used to construct and analyze potentially suitable areas for *L. nigricollis*. Model calibration for the 10 selected environmental variables was performed using kuenm in R v4.3.2, with the regularization multiplier set from 0.5 to 4 in 0.5 increments [35]. The core significance of setting the regularization multiplier range from 0.5 to 4.0 with a step size of 0.5 lies in balancing model complexity and data fitting. A multiplier that is too small may lead to overfitting, while one that is too large could result in underfitting. The 0.5–4.0 range, combined with a step size of 0.5, allows for a comprehensive exploration of the potential optimal parameter range, ensuring the reliability of the model. The MaxEnt model was run with the following parameters: iterations: 500, background points: 10,000, and replicates: 10, with bootstrap chosen as the resampling method for the replicates. In this program, 75% of the distribution points were utilized for model construction, while 25% were reserved for model testing [36]. For the MaxEnt model, the receiver operating characteristic (ROC) curve and area under the curve (AUC) were employed to assess the predictive capability and accuracy (Figure S2), respectively. The omission rate and AICc were used to evaluate the model performance and complexity. The centroid of suitable areas refers to the spatial center of a species’ suitable habitats, and its migration reflects the overall trend of changes in suitable areas. The coordinates of the centroid are calculated in ArcGIS by using the distribution probability of grid cells as weights. The Jenks natural breaks classification and calculated suitable areas were conducted in ArcGIS v10.2. The suitable areas of *L. nigricollis* were divided into four groups: 0–0.094 representing unsuitable area, 0.095–0.203 representing low-suitability area, 0.204–0.403 representing moderate-suitability area, and 0.404–1 representing high-suitability area. To verify the reliability of the classification thresholds, this study compares the thresholds (0.095, 0.204, 0.404) with actual species distribution records: areas with a probability <0.095 showed no occurrence points, while the distribution point percentages for low, medium, and high suitability areas are 8.3%, 31.7%, and 60.0%, respectively. This threshold classification aligns closely with the species’ survival preferences.

## 3. Results

### 3.1. Evaluation of the MaxEnt Model and Dominant Environmental Variables

The lowest value of AICc is approximately 3261, and the omission rate at 5% is 0.05 in this study. The value of AUC is also one of the most important indicators for the Maxent model. After training the data of *Lagria nigricollis* 10 times, the average value of AUC is 0.991 and the standard deviation is 0.001 (Figure S3), indicating that the predictive capability and accuracy of the MaxEnt model are excellent.

The Jackknife method was used to evaluate the importance of environmental variables. The contribution and permutation importance of 10 environmental variables were provided (Table 1). For the contributions of each factor to the model, the top four contributors were the precipitation of the warmest quarter (bio18), the temperature seasonality (bio04), the precipitation seasonality (bio15), and the mean temperature of the coldest quarter (bio11), with contribution rates of 60.7%, 25.1%, 5%, and 4.7%, respectively. According to the permutation importance, bio18, bio11, and bio4 were the dominant factors, with rates of 55.4%, 29.8% and 7.4%, respectively.

**Figure 2:**
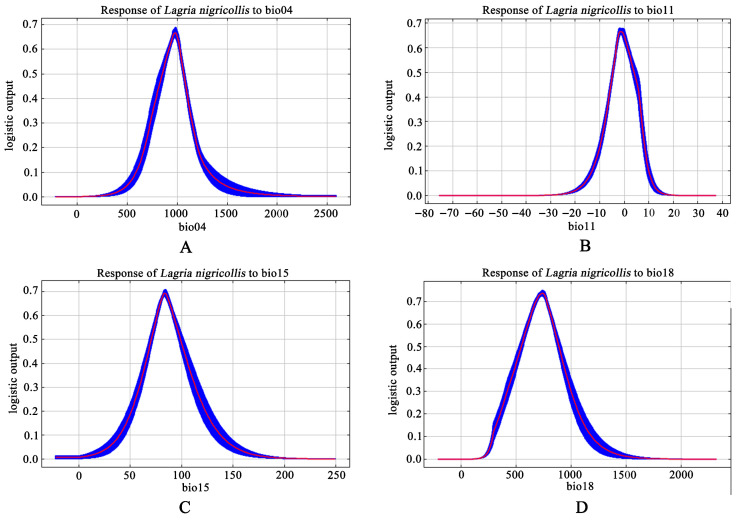
Response curves of the four major environmental variables important for *Lagria nigricollis*.

The response curve intuitively represents the relationship between the environmental variable and the potential distribution probability of *Lagria nigricollis* (Figure 2). For bio18, when the precipitation of the warmest quarter is 736.11 mm, the potential distribution probability of *L*. *nigricollis* reaches its maximum value of 0.75. For bio04, when the standard deviation of temperature seasonality is 983.33, the potential distribution probability reaches its maximum value of 0.66. For bio15, when the standard deviation of precipitation seasonality is 80.64, the potential distribution probability reaches its maximum value of 0.77. For bio11, when the mean temperature of the coldest quarter is −1.72 °C, the potential distribution probability reaches its maximum value of 0.66.

### 3.2. Suitability Area Under Current Condition

**Figure 3:**
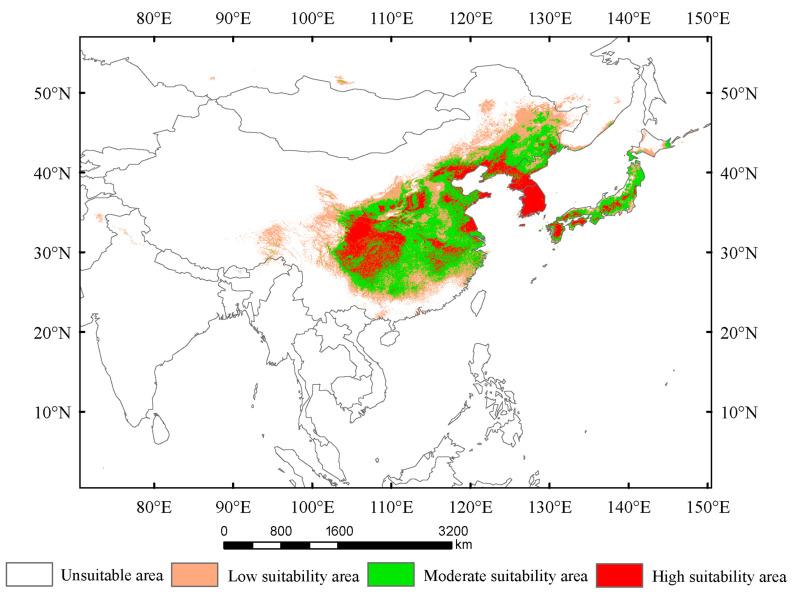
The suitable areas of *Lagria nigricollis* under current climate conditions.

Under current climatic conditions, the total suitable areas of *Lagria nigricollis* are approximately 3.78 × 10^6^ km^2^, which includes high-, moderate-, and low-suitability areas (Figure 3). The high-suitability areas cover approximately 8.44 × 10^5^ km^2^, mainly located in China (North China, Central China), the Korean Peninsula (southern North Korea, South Korea), and Japan (Kyushu, Shikoku, and southern Honshu). The moderate-suitability areas cover approximately 1.64 × 10^6^ km^2^, mainly concentrated in China (North China, Central China, and Northeast China), the Korean Peninsula (Northern North Korea), and Japan (Kyushu, Shikoku, Honshu, and a small part of southern Hokkaido). The low-suitability areas cover approximately 1.29 × 10^6^ km^2^, primarily concentrated in China, with small fragments occurring in North Korea, Japan and Russia.

### 3.3. Suitable Area Under Future Climate Change

**Figure 4:**
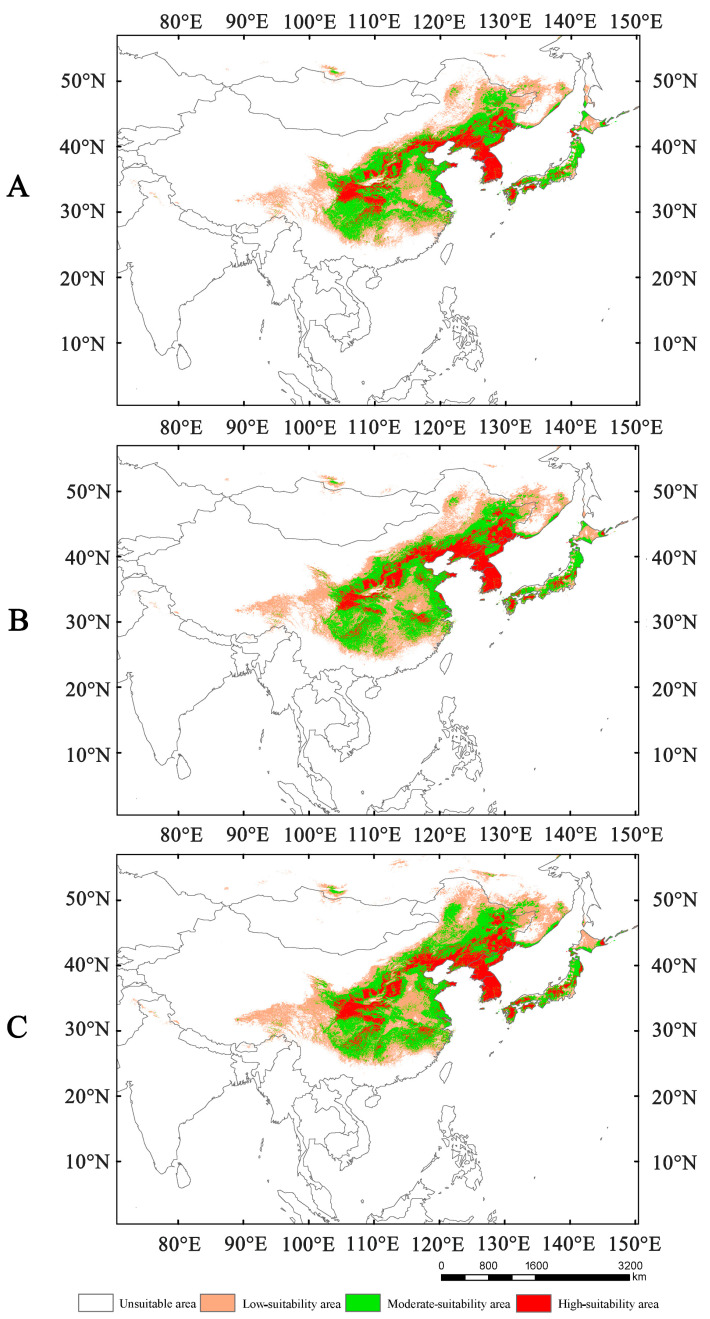
The suitable areas of *Lagria nigricollis* under SSP1-2.6 (**A**), SSP2-4.5 (**B**), and SSP5-8.5 (**C**) in the 2050s.

**Figure 5:**
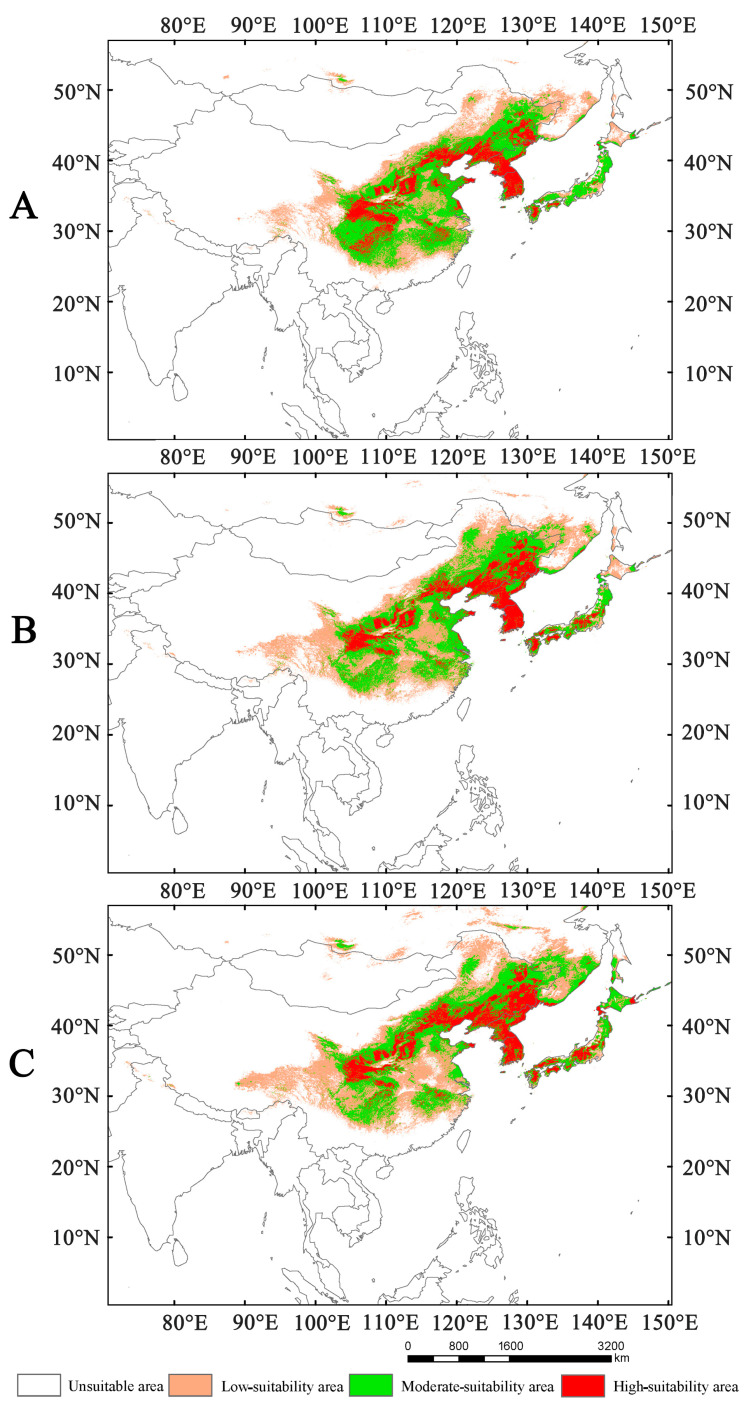
The suitable areas of *Lagria nigricollis* under SSP1-2.6 (**A**), SSP2-4.5 (**B**), and SSP5-8.5 (**C**) in the 2070s.

**Figure 6:**
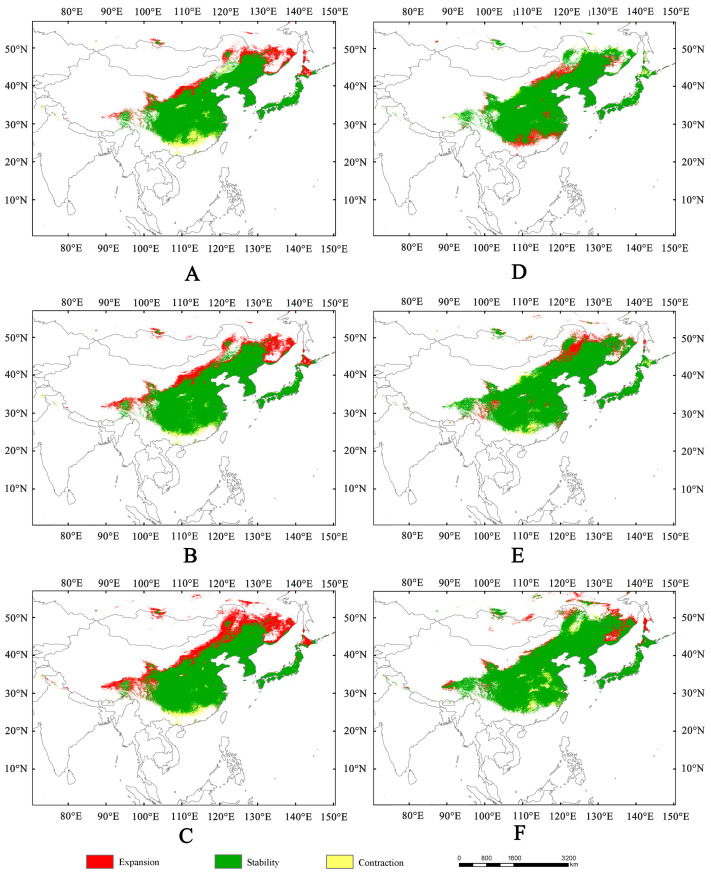
Changes in suitable areas for *Lagria nigricollis* in the 2050s and 2070s. (**A**) Changes in the suitable area under SSP1-2.6 in the 2050s, compared to the current suitable area. (**B**) Changes in the suitable area under SSP2-4.5 in the 2050s, compared to the current suitable area. (**C**) Changes in the suitable area under SSP5-8.5 in the 2050s, compared to the current suitable area. (**D**) Changes in the suitable area under SSP1-2.6 in the 2070s, compared to that in the 2050s. (**E**) Changes in the suitable area under SSP2-4.5 in the 2070s, compared to that in the 2050s. (**F**) Changes in the suitable area under SSP5-8.5 in the 2070s, compared to that in the 2050s.

Compared to the current potential distribution, the suitable areas for *Lagria nigricollis* are projected to expand in the 2050s and 2070s (Table S1). In the 2050s, the suitable areas under SSP1-2.6, SSP2-4.5, and SSP5-8.5 are approximately 4.08 × 10^6^ km^2^, 4.42 × 10^6^ km^2^, and 4.88× 10^6^ km^2^, respectively (Figure 4). During this period, high-, moderate-, and low-suitability areas will expand, alongside increasing levels of greenhouse gas emissions. In 2070s, the suitability areas under SSP1-2.6, SSP2-4.5, and SSP5-8.5 are approximately 4.29 × 10^6^ km^2^, 4.65 × 10^6^ km^2^, and 5.03 × 10^6^ km^2^, respectively (Figure 5). In both the 2050s and 2070s, the suitability areas for *L. nigricollis* show the greatest increase under SSP5-8.5. In the 2050s, the expanded suitable areas are mainly located in the Hengduan Mountains, the junction of the Inner Mongolian region with North China and Northeast China, the southeast of Russia, and the Hokkaido of Japan (Figure 6). In the 2070s, the location of expanded suitable areas changes significantly, but they are primarily concentrated in the northern part of the suitable areas (Figure 6).

### 3.4. Shift in the Centroids

**Figure 7:**
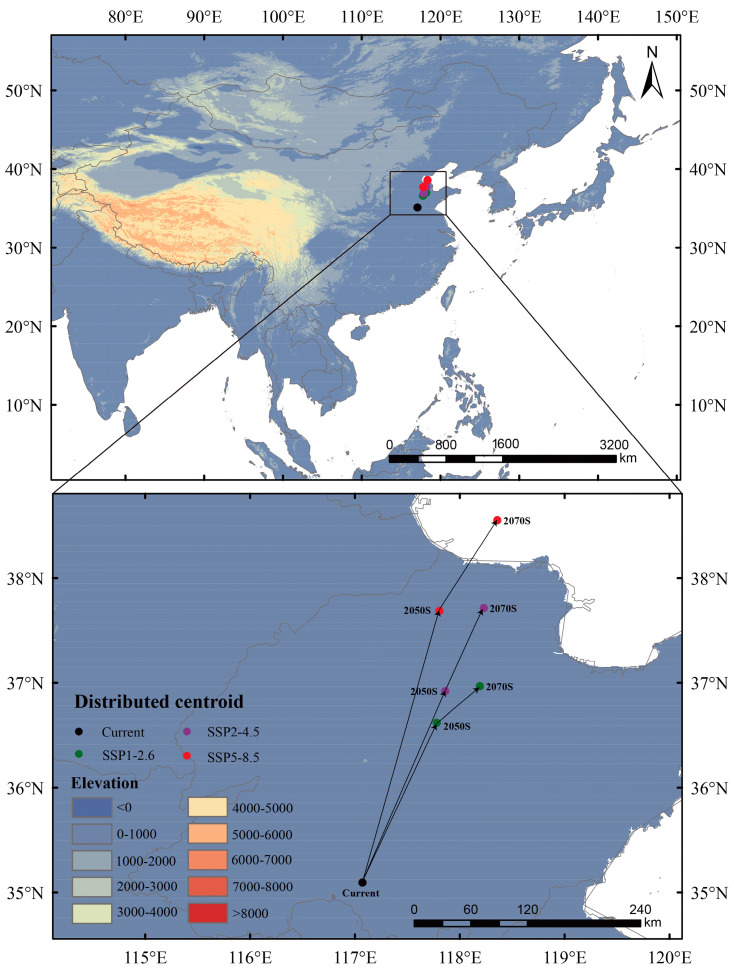
Shift in the centroids of suitable habitats for *Lagria nigricollis* under various climate scenarios.

The centroid of the suitable area will shift with the changes in its distribution. Under current climate conditions, the centroid is located in Yutai County (117.07198° E, 35.09453° N) of Shandong Province, China. In the 2050s and 2070s, the centroids of future suitable areas will shift to the northwest under future climate scenarios (Figure 7). During the same period, the migration distance of the centroid under SSP5-8.5 is the farthest, compared to those under SSP1-2.6 and SSP2-4.5.

## 4. Discussion

The MaxEnt model has been widely used to explore the impact of future climate change on the distribution of insects [37,38,39,40,41]. These research findings have important guiding implications for the prevention and control of future invasive species [37,38,39] and the conservation of rare insects [10,42,43]. For this model, the values of AUC, AICc, and omission rate are three key indicators used to assess the model’s accuracy and complexity. In this study, the high values of these two indicators suggested that the accuracy of the prediction results is very high and that the model fits the data well.

For the environmental variables, bioclimatic and soil factors significantly influence species distribution at a large spatial scale [29]. In this study, the bioclimatic variables, bio18 and bio04, are the most important factors influencing the suitable area of *Lagria nigricollis*, with contribution values significantly higher than those of other variables. This may be due to changes in temperature and precipitation affecting the food source of *L. nigricollis*, thereby influencing its distribution. Regarding soil variables, the contribution of global land cover type is greater than that of elevation and global vegetation, which may be due to the survival of this species being largely constrained by host plants [40]. The precipitation simulation bias of the BCC-CSM2-MR may interfere with the contribution analysis of key variables such as bio18, thereby affecting the assessment of suitable area expansion and migration trends. Additionally, if there is sampling bias in the species occurrence data, it may lead to deviations in the estimation of suitable ranges, particularly affecting prediction accuracy in the marginal areas.

The impact of future climate change on the suitable areas of species is inconsistent [44]. Some studies suggest that the suitable areas of insects will expand [40,45,46,47,48], while others indicate that they will shrink [10,49,50]. Currently, studies using models to explore how Tenebrionidae species respond to climate change are limited, with one species, *Luprops orientalis*, being reported [26]. The results of that study suggested that the suitable areas for *Luprops orientalis* would expand under future climate conditions compared to current conditions.

In this study, the suitable areas of *Lagria nigricollis* under future climate conditions are significantly larger than those under current conditions. Whether in the 2050s or the 2070s, the suitable areas for *L. nigricollis* will gradually expand as the level of greenhouse gas emissions increases. This is inconsistent with the known results for *Luprops orientalis* in the subfamily Lagriinae: in the 2070s, the suitable area of *L. orientalis* is projected to decrease as greenhouse gas emissions increase [26]. As the suitable areas expand northward, the risk of damage to host plants such as mulberry and locust trees in northern regions may increase. Therefore, it is recommended to establish host plant monitoring plots within the suitable areas and deploy pheromone traps for more effective prevention and control. The suitable areas of *Lagria nigricollis* will shift northward under future climate conditions, aligning with the results of most studies [38,51,52]. Given the above, the specific impact on the suitable areas of reported tenebrionid species under future climate change remains inconsistent. Therefore, to better understand how Tenebrionidae species respond to climate change, more species should be studied through modeling.

Based on the locations of highly suitable areas for *Lagria nigricollis* under current climate conditions, the main management regions for this species are concentrated in China (Sichuan, Guizhou, Chongqing, Hubei, Hunan, Gansu, Shaanxi, Shanxi, Hebei, Liaoning, Jiangsu, Anhui, Jiangxi), the Korean Peninsula, and Japan (Kyushu, Shikoku, and the southern part of Honshu) (Figure 3). In the future, to prevent the northward spread of this species, the main control regions will be concentrated in China (the junction of the Inner Mongolia–Xinjiang and Northern China regions), Russia (southeastern Far Eastern Federal District), and Japan (Hokkaido) (Figure 6).

For the results of this study, the authors acknowledge that the model did not incorporate dispersal mechanisms or biotic interactions, both of which may influence the species’ distribution. Furthermore, the distribution of host plants and land use factors was not fully accounted for, which may result in incomplete or less accurate predictions.

## 5. Conclusions

The results of potentially suitable habitats for *Lagria nigricollis* indicate that the precipitation of the warmest quarter and the temperature seasonality are the two most important environmental variables influencing the distribution of *L. nigricollis*. Under current climate conditions, the highly suitable areas for this species are located in China (Sichuan, Guizhou, Chongqing, Hubei, Hunan, Gansu, Shaanxi, Shanxi, Hebei, Liaoning, Jiangsu, Anhui, and Jiangxi), the Korean Peninsula, and Japan (Kyushu, Shikoku, and southern Honshu). In the 2050s and 2070s, the suitable areas will expand and shift northward under SSP1-2.6, SSP2-4.5, and SSP5-8.5, becoming 1.08–1.33 times larger than they are currently.

## Figures and Tables

**Table 1:** The selected 10 environmental variables were used for the MaxEnt model.

Variable	Description	Contribution (100%)	Permutation Importance (100%)
bio18	Precipitation of the warmest quarter	60.7	55.4
bio04	Temperature seasonality	25.1	7.4
bio15	Precipitation seasonality	5	1.4
bio11	Mean temperature of coldest quarter	4.7	29.8
bio19	Precipitation of the coldest quarter	1.5	1.2
gm-lc	Land cover type	1.4	0.4
bio02	Mean daily temperature range	0.7	1.4
bio08	Mean temperature of the wettest quarter	0.5	2.4
elev	Elevation	0.3	0.5
gm-ve	Global land vegetation	0.1	0.1

## Data Availability

The original contributions presented in this study are included in the article/Supplementary Material. Further inquiries can be directed to the corresponding author.

## Supplementary Materials

The following supporting information can be downloaded at https://www.mdpi.com/article/10.3390/insects16121227/s1, Table S1: Statistical analysis of the changes in suitable area under different scenarios; Figure S1: Pearson correlation analysis of environmental variables; Figure S2: The Jackknife test of variables’ importance for the *Lagria nigricollis* using MaxEnt model; Figure S3: The ROC curve for predicting the potential distribution range of *L. nigricollis* based on the MaxEnt model.

## Abbreviations

The following abbreviations are used in this manuscript:

SDMs	species distribution models
*L*. *nigricollis*	*Lagria nigricollis*
MaxEnt	maximum entropy
ROC	receiver operating characteristic
AUC	area under the curve
