# Supplementary material for: Predicted Global Redistribution of Lagria nigricollis (Coleoptera: Tenebrionidae) Under Future Climate Change
Source: Insects. 2025 Dec 3;16(12):1227. doi: 10.3390/insects16121227 (PMC12733788; doi:10.3390/insects16121227)
Supplement: Supplementary file 1 [file insects-16-01227-s001.zip › insects-3947014-supplementary.pdf]

Table S1. Statistical analysis of the changes in suitable area under different scenarios.

Scenarios, times	Expansion (%)	Absence in both (%)	Stability (%)	Contraction (%)	Expansion (km^2)	Absence in both (km^2)	Stability (km^2)	Contraction (km^2)
current-2050s, SSP126	2.46	80.74	15.26	1.54	580108.96	19027989.33	3596448.99	362544.50
current-2050s, SSP245	3.29	79.92	15.93	0.87	774206.89	18833891.41	3753998.46	204995.03
current-2050s, SSP585	5.05	78.15	15.90	0.90	1190319.83	18417778.47	3746688.77	212304.72
2050s-2090s, SSP126	1.63	80.64	17.09	0.63	385255.79	19005278.04	4027990.50	148567.45
2050s-2090s, SSP245	1.63	79.15	18.34	0.87	384527.52	18654358.92	4321996.53	206208.82
2050s-2090s, SSP585	1.63	77.42	19.69	1.25	383907.14	18246176.05	4641519.05	295489.55

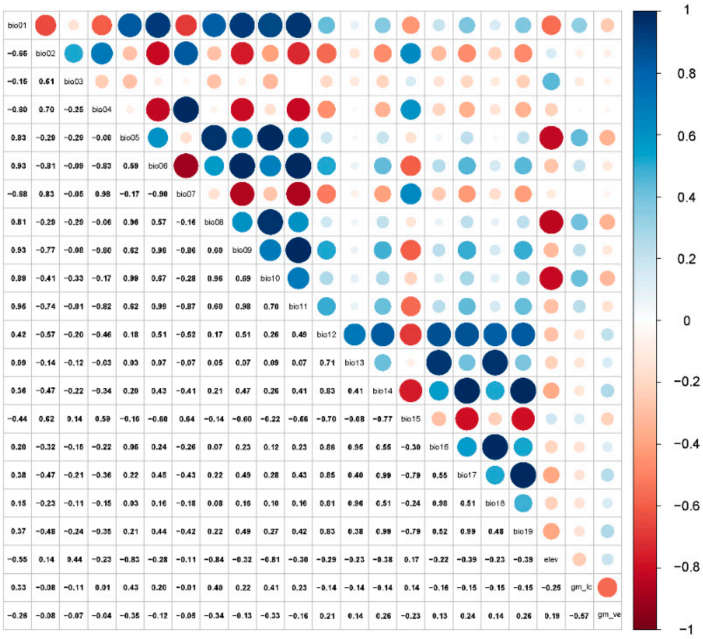

Figure S1. Pearson correlation analysis of environmental variables.

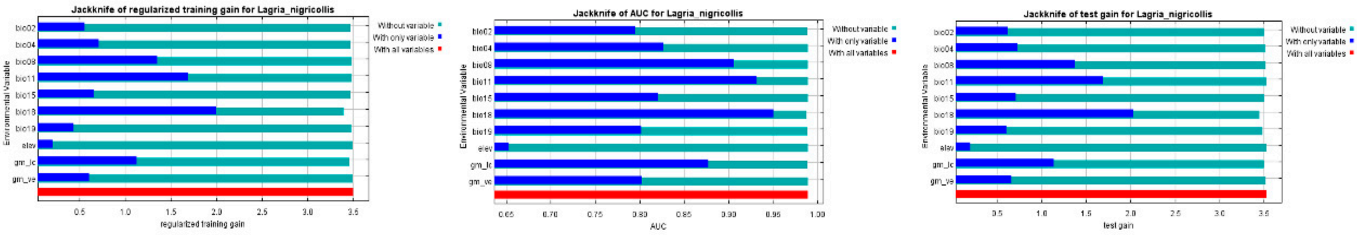

Figure S2. The Jackknife test of variables' importance for the *Lagria nigricollis* using MaxEnt model.

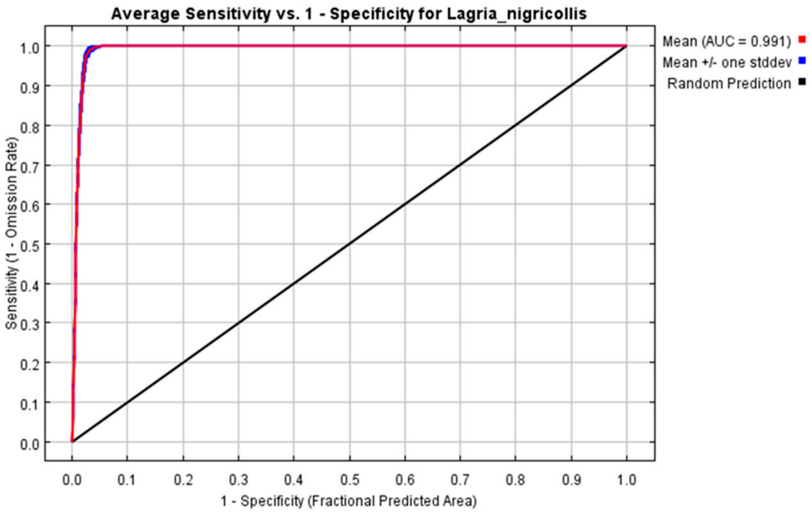

Figure S3. The ROC curve for predicting the potential distribution range of *Lagria nigricollis* based on the MaxEnt model.
